# Editorial: Impact of COVID-19 on the clinical microbiology laboratory: Preparing for the next pandemic

**DOI:** 10.3389/fcimb.2022.1031436

**Published:** 2022-09-20

**Authors:** Sherry Dunbar, Esther Babady, Sanchita Das, Catherine Moore

**Affiliations:** ^1^ Scientific Affairs, Luminex, A DiaSorin Company, Austin, TX, United States; ^2^ Clinical Microbiology Service, Memorial Sloan Kettering Cancer Center, New York, NY, United States; ^3^ Department of Laboratory Medicine, Clinical Center National Institutes of Health, Bethesda, MD, United States; ^4^ Wales Specialist Virology Centre, Public Health Wales NHS Trust, Cardiff, United Kingdom

**Keywords:** COVID-19, SARS-CoV-2, pandemic, clinical laboratory, strategies

Since the first cases of pneumonia caused by the novel coronavirus, SARS-CoV-2, were recognized in December 2019, the world has been consumed by the ongoing COVID-19 pandemic. Early, accurate diagnosis and the ability to differentiate SARS-CoV-2 from other respiratory pathogens remains crucial for rapid clinical intervention and infection control. The clinical diagnostic laboratory is key to these efforts and yet, laboratories and diagnostic manufacturers as well were severely impacted by the rapidly evolving pandemic as never before. In addition to the overwhelming surge in patients with COVID-19, there were few diagnostic tests available at the outset and, with only a fraction of laboratories capable of developing, validating, and running gold standard RT-PCR tests for detection of SARS-CoV-2, the need for commercial tests was quickly apparent. But, as commercial tests became available, laboratories experienced backorders and supply chain issues, not only for SARS-CoV-2 tests, but also for many other routine tests and associated testing supplies, such as swabs, transport media, and extraction reagents. Laboratories experienced staffing shortages as more trained technologists were needed to meet the demand for continuous SARS-CoV-2 testing and staffing was also limited to reduce exposure risk or for quarantine. The laboratory has been required to constantly change and adapt to the evolving pandemic and the need for testing, the type of test needed, and the test demand.

In this Frontiers Research Topic, contributors from diagnostic laboratories in different settings from around the world, as well as diagnostic manufacturers, share their experience with the COVID-19 pandemic, both the common and the unique challenges each faced, as well as mitigation strategies that were implemented to solve issues and achieve success. It is our hope that the experiences and valuable lessons shared here will help laboratories prepare for the future. Improving laboratory preparedness for the next pandemic is an important proactive step for the future of the clinical microbiology laboratory.

Molecular methods, such as RT-qPCR and isothermal amplification, are the gold standard for SARS-CoV-2 diagnosis, thus it is important to understand how these tests may be utilized in different settings. Vindeirinho et al. provide a comprehensive review of the nucleic acid amplification tests (NAATs) and methods available for diagnosis of COVID-19 in the clinical laboratory and at the point of care (POC).

While nucleic acid amplification methods have the distinct advantage of exquisite sensitivity, they cannot differentiate viable from dead organisms. Viral RNA may remain detectable in clinical specimens for a prolonged duration after patient recovery but clinical significance and infectivity in these cases remain unclear. Sung et al. report their research on isolation of SARS-CoV-2 in culture in immunocompromised patients with persistently positive SARS-CoV-2 RT-PCR test results. Of the 20 patients studied, two patients with hematologic malignancies had positive viral cell cultures. More data is needed to determine risk factors for persistent viral shedding and methods to prevent transmission from immunocompromised patients.

Molecular testing is also important for epidemiology and surveillance to monitor the spread and progression of a pandemic. Morris et al. describe the use of large-scale SARS-CoV-2 molecular testing in combination with whole genome sequencing for genomic surveillance in real-time. The extensive laboratory, clinical, and genomic data provided an important resource to help better understand cases of reinfection versus extended RNA shedding and prolonged infections.

As the demand for rapid and high-throughput detection of viral nucleic acid from clinical samples increased, fast and evident inactivation of SARS-CoV-2 is crucial to ensure operator safety during testing. Thom et al. determined efficiency of SARS-CoV-2 inactivation using commercially available lysis buffers on 96-well RNA extraction platforms. They found that methods including both chemical and physical methods inactivated the virus at all titers tested.

Nucleic acid POC testing for SARS-CoV-2 emerged quickly during the pandemic and has been widely used in a variety of settings. Mo et al. reports an expert consensus on SARS-CoV-2 nucleic acid POC testing for China, developed in cooperation by experts in laboratory medicine. Management of the entire process including use cases, biosafety, personnel, verification, quality control, and reporting are described.

Rapid antigen tests are also a common tool for SARS-CoV-2 detection. These tests are relatively fast and inexpensive and have the potential to improve testing capacity in resource-constrained settings. Morales-Jadán et al. conducted a multi-center evaluation of three commercial SARS-CoV-2 rapid antigen tests compared to RT-qPCR in a community setting in Ecuador. The authors found that these tests were adequate for surveillance and detection of infectious individuals in this setting.

Concomitant with detection of SARS-CoV-2 is understanding the host immune response to the infection. Lee et al. evaluated the analytical performance of commercially available surrogate virus neutralization and chemiluminescent assays and determined correlation to SARS-CoV-2 antibody titer by a plaque reduction neutralization test. The chemiluminescent assays had the highest sensitivities and specificities. Measurement of anti-receptor binding domain (RBD) IgG showed the best correlation with the plaque reduction neutralization assay in acute and convalescent phases and correlated to neutralizing activity.

Rivera-Olivero et al. determined the diagnostic performance for seven commercially available serological assays to assess their utility for seroprevalence population studies in South America. There were no statistically significant differences among the assays for anti-SARS-CoV-2 IgG and the tests were deemed acceptable for seroprevalence screening.

Chiu et al. studied humoral, cellular, and cytokine immune responses against SARS-CoV-2 variants in COVID-19 patients to assess correlation with disease severity. The delta and omicron variants were significantly resistant to the humoral immune response generated by individuals infected with the alpha variant. There was significant correlation between disease severity, humoral immune response, and cytokine/chemokine levels, but no evident antibody-dependent enhancement (ADE).

Manufacturers of laboratory reagents and diagnostic tests had to deal with many of the same challenges faced by clinical laboratories. As laboratories were depending on these manufacturers, they had to be agile to quickly develop and meet demand for SARS-CoV-2 tests, while also continuing to supply everything needed for all other laboratory testing that had to continue. Four articles in this collection provide the unique point of view from the diagnostic industry and the challenges they had to face and overcome to help laboratories endure during the pandemic.

Cárdenas and Roger-Dalbert present an industry perspective describing the agility, partnership, and innovation that was critical to respond to the ever-changing needs of caused by the pandemic. The authors point to several issues encountered and recommendations for improvement in the global response to future pandemics.

Thornberg provides an industry perspective from early in the pandemic within a smaller diagnostic manufacturer. He points out that once the decision is made to develop a test, the information needed is sometimes limited to quickly design a robust test that can be rapidly evaluated and moved into manufacturing. Even with an existing platform and consumable to facilitate new assay development, materials such as oligonucleotides, controls, and clinical samples for testing were in short supply. Guidelines implemented to slow the spread of COVID-19 made working in person challenging when also trying to scale-up manufacturing capacity. Due to these challenges, there was considerable evolution in company policies. Health precautions for in-person workers were quickly deployed and teams working from home adapted to virtual platforms in an electronic work environment. This report highlights the effort by the diagnostic community to rise to the need and quickly release high quality testing materials, despite these roadblocks. Each function in the organization plays a critical role and by working together, the shared experience will allow a better response to future rapid-onset health emergencies.

Every manufacturer had to deal with these same or similar issues in some capacity. Das and Dunbar share a perspective describing the challenges faced by the diagnostics industry in general, particularly companies involved in diagnostic assay development and manufacturing. The article presents mitigation strategies employed during the pandemic and provides insights on possible steps to be undertaken to better prepare for future outbreaks.

Challenges faced by the diagnostics industry can be significantly impacted when parts of the operation occur in different locations, different countries, and/or different continents. Tabb et al. provide an account from a diagnostics manufacturer working with R&D teams in Italy and the U.S., with a U.S.-based manufacturing team. Compounding similar challenges in access to raw materials, control materials, clinical samples, and quarantine requirements, the second global hotspot of the pandemic was in Northern Italy where the company corporate headquarters is located. Assay development had to be accelerated even further to address the urgent situation there. Partnering with laboratories in Italy to assist with testing allowed the assay parameters to be finalized one day before Italy was placed on lockdown. This article describes how the many challenges were overcome and the entire company worked side-by-side for accelerated delivery of the assay to clinical labs in Europe, the U.S., and Canada.

## Author contributions

All authors listed have made a substantial, direct, and intellectual contribution to the work and approved it for publication.

## Acknowledgments

We would like to thank all of the authors who contributed to this important Research Topic.

## Conflict of interest

Author SD was employed by Luminex, A DiaSorin Company.

The remaining authors declare that the research was conducted in the absence of any commercial or financial relationships that could be construed as a potential conflict of interest.

## Publisher’s note

All claims expressed in this article are solely those of the authors and do not necessarily represent those of their affiliated organizations, or those of the publisher, the editors and the reviewers. Any product that may be evaluated in this article, or claim that may be made by its manufacturer, is not guaranteed or endorsed by the publisher.

